# Targeting Blimp-1 in T cells results in activation of T-bet-mediated control of immunosuppression in lung cancer

**DOI:** 10.1007/s00262-026-04381-4

**Published:** 2026-04-15

**Authors:** Susetta Finotto, Mircea T. Chiriac, Susanne Krammer, Zuqin Yang, Laura Neurath, Carol I. Geppert, Elvedina Nendel, Sonja Trump, Adriana Geiger, Stefan Wirtz, Sebastian Zundler, Markus F. Neurath

**Affiliations:** 1 Department of Molecular Pneumology, Friedrich Alexander University Erlangen-Nürnberg (FAU), Universitätsklinikum Erlangen, 91054 Erlangen, Germany; 2 Deutsches Zentrum Immuntherapie (DZI), Erlangen, Germany; 3 Bavarian Cancer Research Center (BZKF), Erlangen, Germany; 4 Comprehensive Cancer Center Erlangen-EMN (CCC ER-EMN), Erlangen, Germany; 5 Department of Internal Medicine 1, Friedrich Alexander University Erlangen-Nürnberg (FAU), Universitätsklinikum Erlangen, 91054 Erlangen, Germany; 6 Institute of Pathology, University Hospital, Friedrich-Alexander-Universität Erlangen-Nürnberg, Erlangen, Germany

**Keywords:** T-bet, Blimp-1, Lck, Lung Cancer, Granzyme M, PD1, IFN, IL-2, T regulatory cells, T cells

## Abstract

**Background:**

Lung cancer is the leading cause of cancer-related death in the world. Blimp-1 is a transcriptional repressor that, by interacting with other transcription factors in lymphocytes, regulates their cellular fate.

**Objective:**

In this study, we focused on the role of Blimp-1 in T cells in lung cancer.

**Methods:**

Here, we analyzed, in a murine model of NSCLC, the role of Blimp-1 in T cells after targeting Blimp-1 in T cells expressing lymphocyte-specific-protein tyrosine kinase (Lck), a kinase crucial for T-cell receptor signaling.

**Results:**

We found that mice lacking Blimp1 expression in T cells have reduced lung tumor load, suppressed lung Foxp3+Treg cells in the lung and draining lymph nodes, and induced T-bet+CD4+T effector cells producing IFN-gamma and interleukin-2. Furthermore, RNA sequencing of spleen CD4+T cells showed an induction of Th1 markers, including TNF, IL-2 and interferon type I-related genes, but also PD1. RNA sequencing of spleen CD8+T cells showed induced Tc1 markers, including IL12rb1, CD44, granzyme M and eomesodermin, in the absence of Blimp1. Finally, in the lung of mice with Blimp1 deficiency in T cells, we found an upregulation of CD8+T cells with increased release of cytotoxic mediators able to induce lung tumor cell death.

**Conclusions:**

These data indicate that the tumor microenvironment induces Blimp-1 in immunosuppressive Treg and T effector cells, thereby limiting the therapeutic efficacy of anti-tumor immune responses. Targeting of Blimp-1 in T cells emerges as a novel concept to suppress immune evasion in lung cancer by regulating CD4+, CD8+and Treg function in the lung.

**Supplementary Information:**

The online version contains supplementary material available at 10.1007/s00262-026-04381-4.

## Introduction

B-lymphocyte-induced maturation protein 1 (BLIMP1) is a transcription repressor crucial in controlling the terminal differentiation of antibody-secreting cells (ASCs). In addition, Blimp-1 has an important role in mantaining the homeostasis of effector T cells. Non-small cell lung cancer (NSCLC) is a frequently diagnosed cancer type and a leading cause of cancer-related death [1, 2]. The treatment landscape of resectable NSCLC has been enhanced for patients with NSCLC following the approval of systemic treatments combined with chemotherapy [3, 4].

Many clinical challenges persist in the face of these advances. For example, factors such as abnormal antigen presentation, functional gene mutations, and the tumor microenvironment can contribute to treatment resistance. The mechanisms of acquired resistance to these therapies are under investigation [3].

We and others have reported increased immunosuppression in the lungs of patients with NSCLC. Specifically, in a cohort of patients with NSCLC, we demonstrated an increased number of Foxp3⁺ T regulatory cells infiltrating the tumor [2]. In addition, by using murine models of NSCLC, we demonstrated the presence of tumor-infiltrating immunosuppressive T-bet⁺Foxp3⁺ and PD1⁺ T cells, which represent an additional TGF-beta-mediated mechanism by which the tumor evades the immune system locally. By contrast to Foxp3, T-bet is a transcription factor that controls tumor cells by driving T-cell-mediated cancer cell death [2–4]. Moreover, the effector T regulatory cells express Blimp1 encoded by *Prdm1* [5].

Tumor-infiltrating lymphocyte (TIL) Blimp-1-expressing T regulatory cells have been proposed as markers for outcome in some patients with tumors [6]. In line with this, Blimp1 has been identified as an important transcription factor required for the lineage stability and suppressive activity of Foxp3⁺ T regulatory cells [7]. Thus, in this manuscript, we sought to further investigate the role of Blimp1 in tumor-infiltrating lymphocytes (TILs) and Foxp3⁺ T regulatory cells to better understand how T-bet and Blimp1 regulate each other in T cells in the tumor microenvironment (TME).

## Methods

### Mice and genotyping

All mice were bred and maintained under specific-pathogen-free (SPF) conditions in the local animal facility (University Hospital Erlangen, Erlangen, Germany). All experiments were performed in accordance with the German and European laws for animal protection and were approved by the local ethics committees of the Regierung Unterfranken (Az 55.2-2532-2-1286-20). The *Blimp1*^fl/fl^, *Blimp1*^fl/fl^ *GFP, LckCre*, LckCre-GFP, *T-bet deficient* mice (generous gift from Prof. Laurie H. Glimcher) and wild-type mice utilized in this study share a common genetic background of C57BL/6. The *Blimp1*^fl/fl^, *Blimp1*^fl/fl^ *GFP, LckCre*, and *LckCre*-GFP *Blimp1*^fl/fl^ mice (genotyped with the following primers: Com F 5’ TGAGTAGTCACAGAGTACCCA-3’; wt 5’-GCGGAATTCATTTAATCACCCA-3’; KO 5’-GGCAAGATCAAGTATGAGTGC-3’), were generously provided by researcher Professor Klaas P.J.M. van Gisbergen from Dept of Hematopoiesis, Sanquin Research and Landsteiner Laboratory Amsterdam UMC, University of Amsterdam, Amsterdam, Netherlands [8].

This transgenic strain expresses Cre under the control of the *Lck* (lymphocyte protein tyrosine kinase) promoter, enabling thymocyte-specific excision of *loxP*-flanked sequences of Blimp1. The *Lck* gene is primarily expressed by T lymphocytes, and it plays a key role in the selection and maturation of developing T cells in the thymus and in the activity of mature T cells.

The conditional knockout *Blimp1*^fl/fl^x *LckCre* mice were generated by crossing *Blimp1*^fl/fl^ with *LckCre* mice (Blimp ^fl/fl^ x B6.Cg-Tg(*Lckcre*)548Jxm/J Strain #:003802 RRID:IMSR_JAX:003802Common Name: LckCre 548-O). All experiments were conducted using mice aged between six and eight weeks. The study employed both male and female mice.

### Murine model of experimental lung tumors

At our laboratories, we established an experimental lung tumor model of intravenous injection of LL/2-Luc-M38 (LL/2) lung adenocarcinoma cells in commercially obtained C57BL/6 J wild-type mice. LL/2 cells were cultured in DMEM (Dulbecco`s modified eagle medium high glucose, anprotec, Cat# AC-LM-0012), supplemented with 10% FCS, 1% pen/strep, and 1% L-Glu at 37 °C and 5% CO_2_. For tumor induction in eight-weeks-old female or male mice (obtained from Elevage Janvier), 5 × 10^5^ LL/2 cells were injected intravenously (i. v., tail vain) to form organ-specific tumor foci in the lung. Animals were kept under antigen-free conditions in the animal facility, and experiments were performed in accordance with the German and European laws for animal protection. The LL/2-M38-Luc cell line (purchased from Caliper Life Sciences) expresses the enzyme luciferase through an introduced vector (resistant to Geneticin G418) and originates from the murine lung tumor Lewis Lung Carcinoma (LLC; ATCC#: CRL-1642), which was isolated from C57BL/6 wild-type mice. The cell line was further sequenced via a DNA barcoding system at the DSMZ Leibniz –Institute before performing these experiments. By intraperitoneal injection of luciferin at the end of the experiment, bioluminescence reaction takes place within the LL/2 cells accompanied by release of photons, which was detected using the in vivo imaging system (IVIS).

For lung tumor induction, 5 × 10^5^ cells resuspended in 200 µl DMEM medium (without supplements) were injected into the tail vein of 6–8 weeks old female mice. At the indicated time points, mice were weighed and injected intraperitoneally (i.p.) with luciferin (0.15 mg per 1 g body weight; Promega, Cat#P1043). Luciferase activity was measured after 20 min by the IVIS Spectrum in vivo imaging System (PerkinElmer), as previously described [9]. Briefly, mice were anaesthetized using isoflurane and luciferase activity was measured by detecting luminescence intensity (photons per second). Analyses were performed in a logarithmic scale mode. Mice were sacrificed at days 14 or 15 after tumor cell injection. The end of the experiment was chosen depending on the fitness of the mice, as longer time courses resulted in markedly augmented tumor burden.

For the in vivo determination of the tumor burden, mice were injected i. p. with luciferin (150 µg per 1 g body weight diluted in 10 µl PBS) the day before the end of the experiment. After incubation for 15 min and narcotization for 5 min, bioluminescence signals were detected via in vivo imaging system (IVIS; Perkin Elmer). The analysis was done with the related software Living Image® 4.7.3 (Perkin Elmer). Overlays of bioluminescence measurements and photographic images were used to determine the average radiancy within defined regions of the thorax areas. Background signals were specified for normalization of all images.

### Organ isolation

After euthanizing the mice, the lungs and spleen were aseptically removed. The thorax was disinfected and laterally opened on both sides to expose the lungs. A portion of the lung tissue was placed into a specialized embedding cassette and fixed in a 4% formaldehyde solution for subsequent histological analysis. Another piece of lung tissue was stored in a 1.5-ml Eppendorf tube at − 80 °C for future protein or RNA analysis. The remaining lung tissue was transferred to 5 ml RPMI medium with 1% penicillin–streptomycin for immediate isolation of lung cells.

To remove the spleen, the left flank of the mouse was disinfected and the skin was removed with scissors to expose the spleen. The entire spleen was then carefully excised and placed in 5 ml RPMI medium with 1% penicillin–streptomycin on ice until further processing.

### Isolation and culture of total lung and splenic cells

Under sterile conditions and following a standardized protocol, total cells were isolated from lung and spleen tissues. Lung tissue was minced, then gently shaken in 10 ml of collagenase (Sigma-Aldrich, München)/DNase (Roche, Mannheim) solution for 45 min at 37 °C. After enzymatic digestion, the cells were separated by homogenizing the tissue through a 40-μm cell strainer with a syringe plunger. The cell suspension was centrifuged (1500 rpm, 10 min, 4 °C), and erythrocytes were lysed using 10 ml ACK lysis buffer, followed by another centrifugation (1500 rpm, 5 min, 4 °C). The supernatant was discarded, and the cells were washed with 10 ml PBS. Slow pipetting and a horizontal pipette position helped to remove remaining fat from the cell suspension. After another centrifugation (1500 rpm, 5 min, 4 °C), the cells were resuspended in 10 ml PBS and counted using a Neubauer chamber and Trypan blue. The same procedure was used for spleen cells, omitting the enzymatic digestion step. The tissue was immediately pushed through the cell strainer. All subsequent steps were identical to those for lung cells.

For lung cell or splenic cell culture with aCD3/28 antibodies, the plate was pre-coated with 0.5 µg/ml αCD3 (Biolegend) in 0.1 M sodium bicarbonate buffer (Carl Roth GmbH & Co. KG, Karlsruhe) for 1h at 37 °C. Post-PBS wash, cells were cultured in R10 medium (10% Fetal bovine serum, 1% L-Glutamine, 1% penicillin–streptomycin in RPMI 1640) with 1 µg/ml αCD28 (Biolegend) added. After 48h cell culture at 37 °C, 5% CO2, the supernatants and cells were harvested. For the splenic CD4+T cells cultured for 6 days, 5 ng/mL of IL-2 was supplemented every other day.

Isolation of CD8+ and CD8- lung T cells for cytotoxic assay was performed by using CD8a+T-cell isolation kit (mouse Cat 130–096-543, Macs, Miltenyi) in accordance to the manufacturer’s instructions.

### Splenic CD8+and CD4+T-cell sorting

For the sorting of splenic CD8+ and CD4+T cells, the procedure began with the blocking of non-specific binding in total isolated splenic cells. Mouse TruStain FcX™ Antibody (anti-mouse CD16/32, BioLegend, Cat#101,320) was used at a 1:100 dilution for 10 min at RT in the absence of light. After blocking, the cells were incubated for 25 min in a 500-μL MasterMix containing CD3 PE, CD4 BV421, and CD8-APC-Cy7 antibodies **(**Table S1**)** at RT. The staining process was completed with FACS buffer, followed by centrifugation to remove the supernatant. Subsequently, the cells were resuspended in FACS buffer, preparing them for sorting at the core unit for cell sorting at Friedrich-Alexander-Universität (FAU) in Erlangen. The sorted cells were further use for RNA isolation and analysis. Alternatively, CD4+ or CD8+ T cells for cell culture were carried out using MACS sorting with mouse CD4 (L3T4) beads (Miltenyi Biotec) and mouse CD8 MicroBeads UltraPure (Miltenyi Biotec), respectively. Initially, CD8+T cells were isolated, followed by the isolation of CD4+T cells from the CD8-negative fraction. These isolation procedures strictly adhered to the manufacturer’s instructions.

### Hematoxylin and eosin staining on murine paraffin-embedded lung sections

The lung lobes were excised, fixed in a 10% formalin-PBS solution, dehydrated, and embedded in paraffin. Five-micrometer-thick sections of lung tissue from paraffin blocks were stained with hematoxylin and eosin for the purpose of visualizing lung tumors. Then, the stained slides were scanned using a digital slide scanner (Scan 150, 3D Histech Ltd.). Subsequently, the slide images were analyzed using the CaseViewer software (Version 2.0, 3D Histech Ltd). The hematoxylin and eosin staining was conducted in collaboration with the Institute of Pathology at the University Hospital Erlangen.

### Tumor load detection

At least 6–9 sections taken at 3 different level of the lung were stained with hematoxylin and eosin (H&E) and digitalized in the Digital Pathology department (Dr.P.D. C.I.Geppert’s Team) using a slide scanner (Scan 150, 3D Histech Ltd). The analysis of the tumor burden within the lung tissue sections was done by the pathologist (C.I.G) in a blinded fashion.

### Bioluminescence cytotoxic detection assay

For the purpose of conducting a cytotoxic analysis of total lung cell supernatant, LL/2-luc-M38 cells were seeded at a density of approximately 7 × 10^3^ cells per well in 96-well white-walled plates (Thermo Fisher Scientific, Waltham, MA, USA, Cat#165,306) and cultured for 24h at 37 °C in a humidified atmosphere of 5% CO₂. A standard curve in quadruplicate with increasing concentration of cells starting on the upper left hand side and proceeding vertically down of the plate with 0, 875, 1750, 3500, 7000, 14,000, and 28,000 cells. Subsequently, the supernatant was replaced after 24h of cell culture. Consequently, the supernatant was discarded, and the cells were washed with 100 μl of PBS. The supernatant from the cell culture and the cells were cultured with the new supernatant for 24h in quadruplicates for each cell culture condition. After 24h, the culture medium was replaced with 100 μl of luciferin (1500 μg/ml, Promega, Cat#P1043) diluted 1:10 in PBS, and the cells were incubated for 25 min at 37 °C in 5% CO2. Bioluminescence measurements were conducted with a counting time of 0.5s at room temperature using a Centro XS3 LB 960 plate-reading luminometer (Berthold Technologies, Bad Wildbad, Germany). Following the completion of the measurements, the mean of the quadruplicates was calculated.

### RNA isolation and RNAseq

To study the transcriptional landscape, total RNA was extracted from FACS sorted spleen CD4+and CD8+T cells, preserved in RNA stabilization solution (RLT buffer) and stored at − 80 °C. Quality control and sequencing were conducted by Novogene Corporation Inc. RNA integrity was checked using an Agilent Bioanalyzer, excluding samples with an RNA Integrity Number (RIN) below 7. Library preparation involved enriching mRNA using poly-T oligo-attached magnetic beads, followed by mRNA fragmentation and cDNA synthesis using random hexamer primers and dTTP for a non-directional library. Library quality was assessed using Qubit, real-time PCR, and Bioanalyzer. Sequencing was performed on Illumina platforms. Raw sequencing data underwent quality control using FastQC, including adapter and low-quality base trimming with Trimmomatic. Reads were aligned to the mouse reference genome (Mus Musculus, GRCm39/mm39).

### Flow cytometric analysis of murine cells

Flow cytometric analysis for surface antigen expression was conducted by seeding 400,000 cells in a 96-well plate (U-bottom) followed by centrifugation (Thermo Fisher Scientific, Megafuge™ 16 Universal-Zentrifuge, Cat# 75,004,270). All centrifugation steps were conducted at 1 min at 2000 rotations per minute at 4 degrees Celsius. Subsequently, the supernatant was discarded, and the cells were incubated with anti-CD16/CD32 (1:100, BD Biosciences, Cat# 553,142) mAb for a minimum of five minutes at 4 °C in the dark to prevent non-specific binding of immunoglobulins to Fc receptors.

The cells were then washed with 150 µl FACS buffer (PBS+2% FCS, Sigma-Aldrich, Cat# S0615) and subsequently centrifuged. Subsequently, the cells were incubated with a 50-µl master mix solution of surface antibodies against various surface antigens for 20 min at 4 °C in the dark. Subsequently, the cells were washed with 150 µl FACS buffer and centrifuged. Intracellular staining was conducted by resuspending cells in 100 µl of FoxP3 Fixation/Permeabilization Reagent (Thermo Fisher Scientific, Cat# 00–5523-00) for 30 min at room temperature in the dark, followed by washing with 200 µl permeabilization buffer (1 part Thermo Fisher Scientific Permeabilization Buffer (10X), Cat# 00–8333-56 and 9 parts Millipore-H2O) for two times. After, intracellular epitopes were stained by the addition of 50 µl of the antibody mix and incubation of the cells for 30 min at room temperature in the dark. The cells were then centrifuged and washed twice with 200 µl permeabilization buffer. Finally, the cells were centrifuged once more and resuspended in 100 µl FACS buffer. The stained cells were then analyzed by flow cytometry (BD FACS Canto 2, or Symphony BD Biosciences, Heidelberg). The antibodies utilized in this study for human and murine FACS analysis are listed in Table S1. The data sets were analyzed using FlowJo v10.2 software (FlowJo, LLC, OR, USA) and Caluza.

### Enzyme-linked immunosorbent assay

The enzyme-linked immunosorbent assay (ELISA) technique was employed to analyze the cytokine concentration in cell culture supernatant, with the procedure conducted in accordance with the manufacturer’s instructions. The BD OptEIA kit (555 138, BD Biosciences, Heidelberg) was used for mouse IFN-γ detection according to the manufacturer’s recommendation.

### Multiplex cytokine assays

To determine the protein concentration of IL-2, IL-6, IL-10 in the medium supernatant of murine cell cultures, the LEGENDplex™ murine Th Cytokine Panel (12-plex), Cat# 741,044, was used in accordance with the enclosed protocols. For this purpose, a mixture of 5 µl assay buffer, 5 µl detection antibody and 5 µl capture beads was pipetted into each well of the enclosed V-bottom plates, which was supplied with 5 µl of the respective sample. After 2h of incubation at room temperature in the dark, 5 µl of the streptavidin-PE solution were added to each well and incubated for 30 min. 150 µl wash buffer were added to each well and the well plates were centrifuged at 2500 rpm for 5 min at RT. The supernatant was then carefully removed and the samples were taken up in 100 µl wash buffer for immediate analysis at the flow cytometer. A standard series for concentration determination was prepared according to the kit protocol.

### Quantitative real-time PCR (qPCR)

Total RNA was extracted from frozen tissue samples or from cell suspension samples using the Qiazol Lysis® Reagent (QIAGEN, Cat#79,306) according to the manufacturer’s instructions. One microgram of the resulting RNA was reverse transcribed into cDNA via the RevertAid™ First Strand cDNA Synthesis Kit (ThermoFisher Scientific, Cat#K1622) according to the manufacturer’s protocol. Each quantitative polymerase chain reaction (qPCR) reaction mixture contained 15 ng of cDNA, 300 nM of transcript-specific forward and reverse primers, and iTaq Universal SYBR Green Supermix (Bio-Rad Laboratories, Cat# 1,725,124) in a total volume of 20 µl. The qPCR primers were procured from Eurofins-MWG-Operon (Ebersberg, Germany). The primer sequences for murine qPCR analysis are presented in the supplementary **Table S2**. The reactions were conducted for 50 cycles, with an initial activation for 2 min at 98 °C, denaturation for 5 min at 95 °C, and hybridization and elongation for 10 min at 60 °C. Quantitative polymerase chain reaction (qPCR) reactions were performed using the CFX-96 Real-Time PCR Detection System (BIO-RAD, Munich, Germany) and analyzed via the CFX Manager Software. The relative expression level of specific transcripts was calculated using the relative quantification 2^−ΔΔCT^ method with respect to the internal standard HPRT. Data with ΔCq values higher than 35 were excluded.

### Statistical analysis

In this study, cell percentages were accurately determined using FlowJo (version 10.9) or Kaluza software, with further statistical analysis performed in GraphPad Prism 9, presenting results as mean ± SEM. For two-group comparisons, the unpaired Student’s two-tailed T-test or the Mann–Whitney test for normally and non-normally distributed data respectively were applied. One-way ANOVA was used for analyzing multiple groups based on a single independent variable, while two-way ANOVA was employed for data involving two independent variables, including their interaction effects. Additionally, correlations were discerned through simple linear regression. A P-value below 0.05 signifies statistical significance. RNA-sequencing data were analyzed using DESeq2 for the identification of differentially expressed genes, with gene enrichment analysis and visualization conducted in R, ensuring a comprehensive and robust statistical evaluation.

## Results

### Targeted deletion of Blimp1 in T cells reduces lung cancer growth in an experimental model of NSCLC

**Fig. 1:**
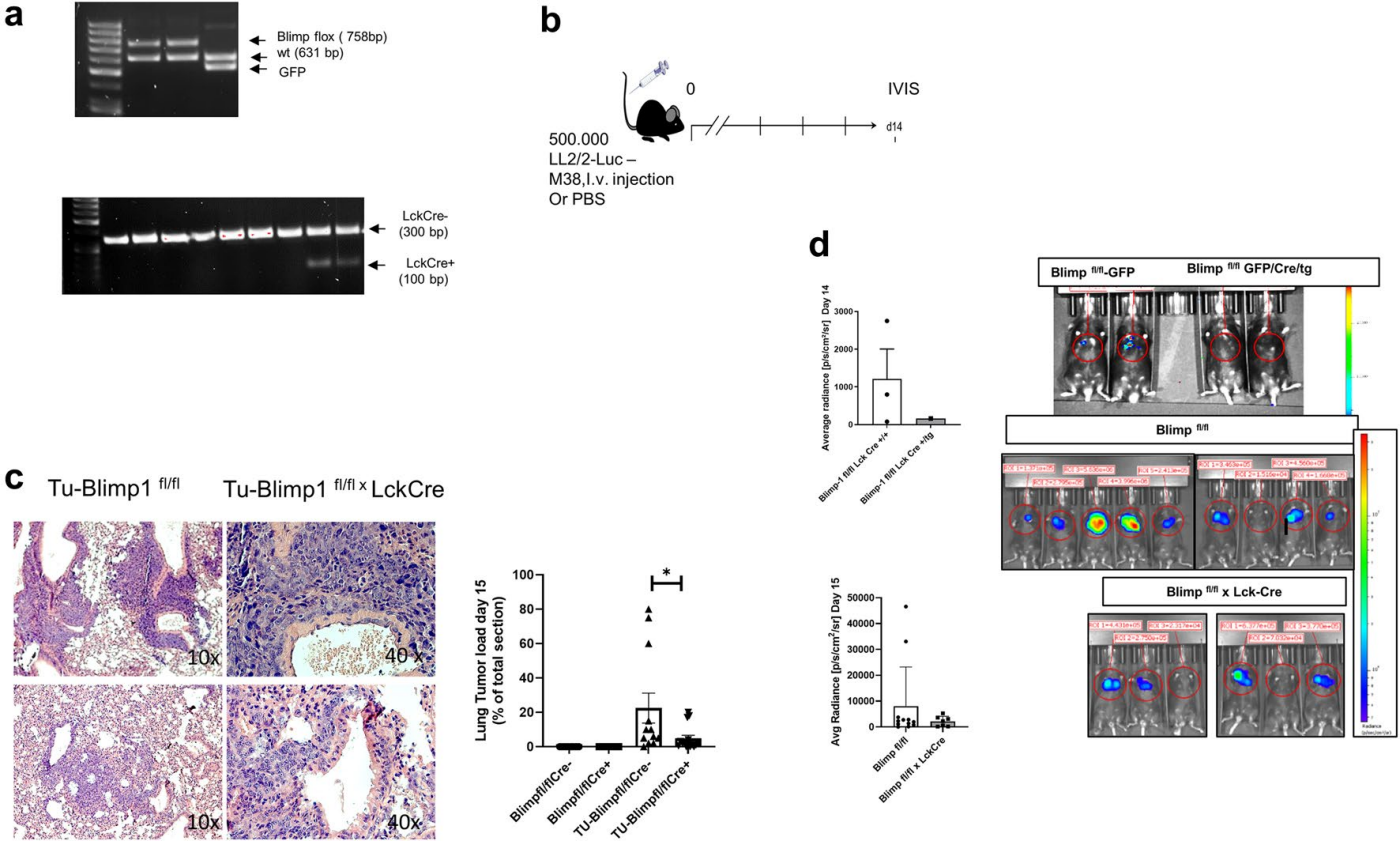
Targeted deletion of Blimp-1 in T cells in Blimp-1.^fl/fl^-LckCre mice results in decreased tumor load as compared to controls*. a*. Genotyping of Blimp-1 flox (fl)/fl -LCK Cre mice. **b.** Experimental design of the NSCLC model used. Mice were analyzed after intravenous injection of LL/2-luc-M38 cells **c**. Quantitative analysis of histopathological data in Blimp-1-proficient and Blimp-1-deficient animals. Histological sections of the lung stained with H&E. Comparison between 2 groups was performed with the Mann–Whitney test (*n* = 12;4;12;15; *p* = 0.0279) **d**. IVIS analysis at day 14 (*n* = 3, 1) and at day 15 (*n* = 12, 7), respectively. Comparison between 2 groups was performed with the Mann–Whitney test: lower panel *p* = 0.90

To investigate the function of Blimp-1 in T cells in lung cancer under in vivo conditions, we utilized a mouse line with a targeted deletion of this protein in T cells **(**Figs. 1** a,b, Supplementary Fig. 1)**. The Blimp^fl/fl^ LckCre+line was examined in comparison to Blimp^fl/fl^LckCre- controls in a murine model of lung tumorigenesis induced by LL/2 luc cell transfer, with tumor load determined by in vivo bioluminescence imaging (IVIS). Histological analysis demonstrated a significant reduction in lung cancer load at day 15 in mice lacking Blimp-1 in T cells in comparison to controls **(**Fig. 1c**),** suggesting that Blimp-1 expression in T cells controls cancer growth in vivo. Moreover, the histologic pictures showed increased apoptotic tumor cells in the absence of Blimp1 in T cells in the lung.

Moreover, by IVIS analysis, a trend toward lower tumor growth was noted at day 14 in the knockout mice as compared to control mice **(**Fig. 1d**)**. This indicates that Blimp-1 expression in T cells controls the growth of established tumor lesions rather than initial tumor development. Moreover, in vivo imaging using IVIS revealed that conditional Blimp-1 knockout mice exhibited markedly reduced tumor loads, although not statistically significant, at day 15 after tumor cell injection compared to control Blimp^fl/flx^ LckCre mice. There was no difference in body weight between the two lines **(Supplementary Figure S2)**, however. In addition, we terminated the experiments when all mice were still alive, and thus no comparison of survival could be made.

### Targeted deletion of Blimp-1 in T cells results in induction of CD4+T-bet+T cells in the lung of tumor-bearing mice

**Fig. 2:**
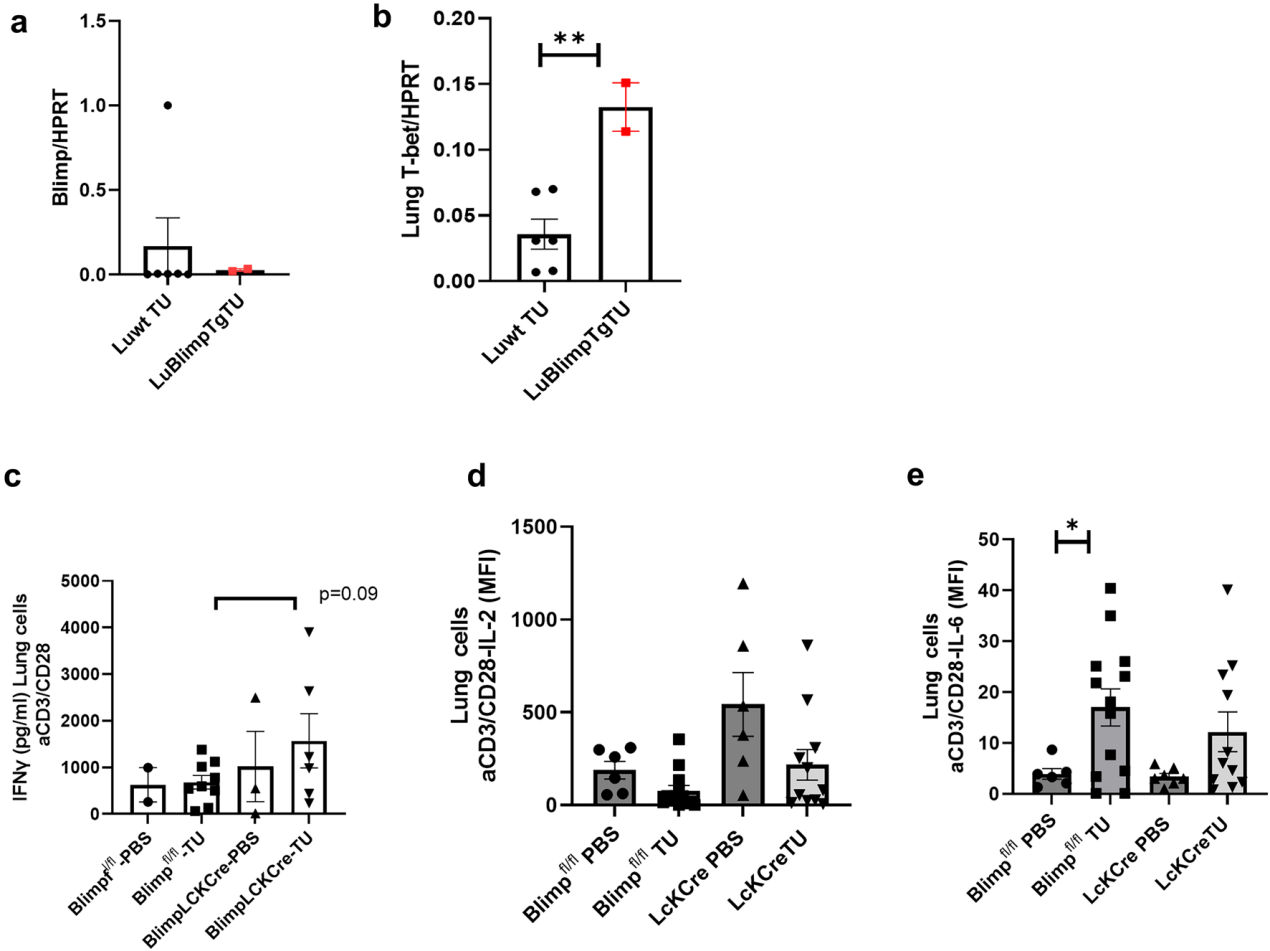
Targeted deletion of Blimp-1 in T cells in Blimp-1^ fl/fl^-LckCre mice results in induction of T-bet mRNA and IL-2 production in the lung in a model of NSCLC, **a**. Gene expression analysis of Blimp-1/HPRT **b.** T-bet/HPRT mRNA expression in the lung (*n* = 6, 2). **c**. IFN-*γ* by ELISA (*n* = 2;9;3;6) and **d.** IL-2 (*n* = 6;13;3;15); *p* = 0.015 and **e.** IL-6 (*n* = 6; 13,7,11); *p *= 0.03 by multiplex analysis in the supernatant of anti-CD3/CD28-stimulated lung cell cultures. Unpaired T-test. Data are expressed as mean values ± SEM

We proceeded to determine whether targeting Blimp-1 function in T cells would destabilize Treg cell function, thereby allowing for the expansion of T-bet-expressing, IFN-*γ*-producing Th1 cells in the immunosuppressive tumor microenvironment in NSCLC. Consequently, first we analyzed and confirmed Blimp1 mRNA reduction in the lung of Blimp-1 knockout mice (Fig. 2a). We next examined lung T-bet mRNA expression. Here, we found upregulated T-bet levels in the lung of Blimp-1 conditional knockout mice as compared to controls (Fig. 2b**).** Then, we examined by ELISA IFN-*γ* released by lung cells from tumor-bearing mice with experimental NSCLC following stimulation with anti-CD3 and anti-CD28 antibodies. We observed an increase by trend in IFN-*γ* levels in the supernatant of lung cells isolated from tumor-bearing mice lacking Blimp-1 in T cells in comparison to controls (Fig. 2c). By using multiplex analysis, IL-2 was found to be significantly induced in Blimp-1-LckCre mice bearing tumor as compared to mice proficient in Blimp-1 in T cells (Fig. 2d). Moreover, we looked at IL-6, a proinflammatory cytokine with a role in cancer [10], and found that IL-6 levels were significantly upregulated when Blimp-1 was expressed in T cells in the experimental tumor setting (Fig. 2e).

**Fig. 3:**
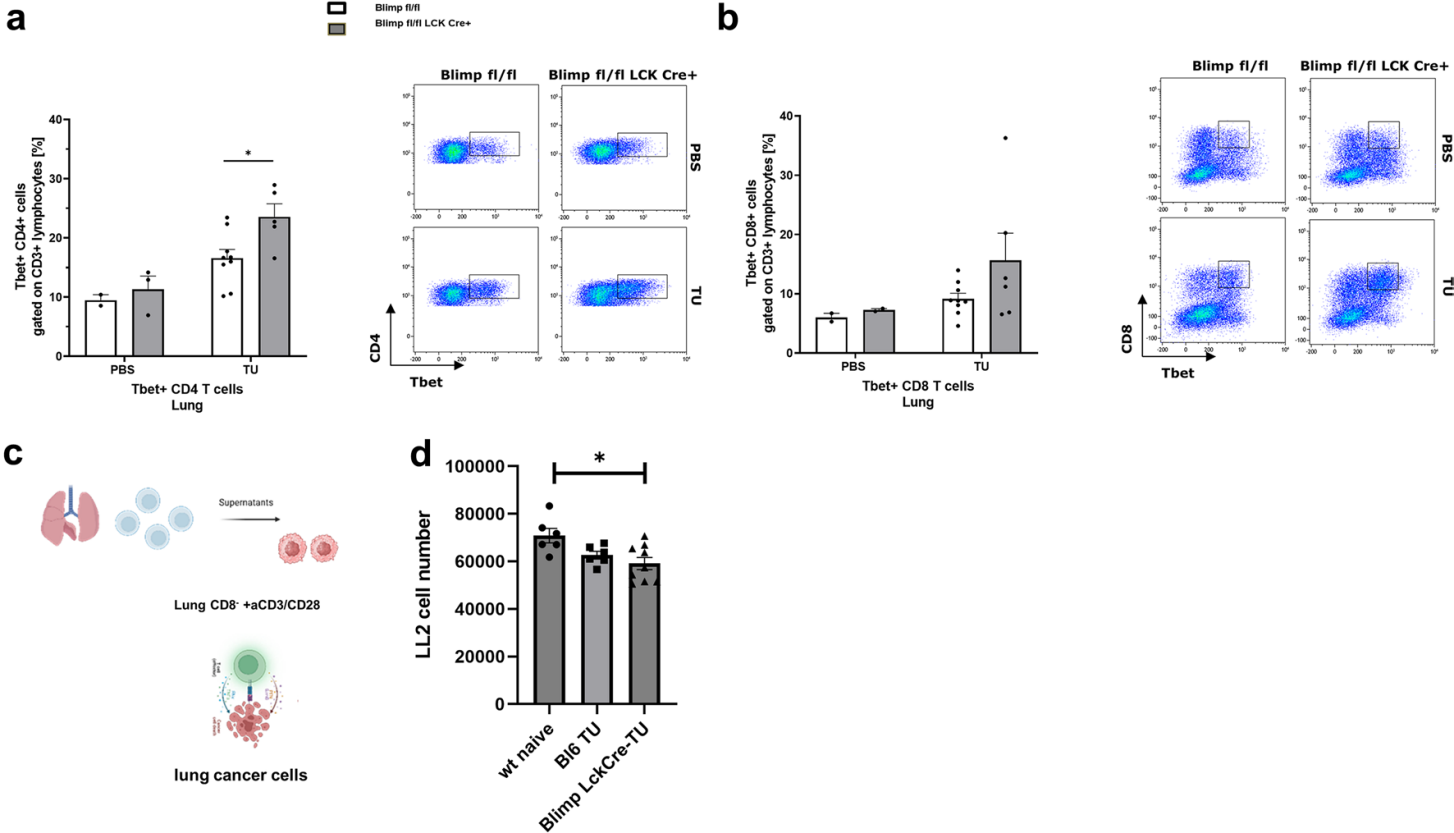
Targeted deletion of Blimp-1 in T cells results in induction of T-bet+CD4+T cells **a.** Flow cytometry analysis of T-bet+CD4 T cells and **b.** CD8+T-bet+T cells in the lung (*n* = 2, 3, 9, 6) of Blimp-1 flox (fl/fl) -and Blimp-1 flox (fl/fl) -LCK Cre mice. **c.** The experimental design of the cytotoxic assay employs the same tumor cell line that was used to induce tumors in mice. Hereby, 30% of the cell supernatants diluted in medium (30% conditioned medium) was analyzed. Total lung cells were sorted by depleting CD8+T cells. The lung CD8- T cells were cultured with anti-CD3-anti-CD28 antibodies and supernatants were diluted to obtain 30% conditioned medium which was analyzed in the cytotoxic assay. (*p* = 0.0112). Unpaired T-test. The different groups are indicated. Data are expressed as mean values ± SEM. Significance was calculated by student’s t-test or ordinary two-way ANOVA with post-hoc Šidák’s multiple comparisons test

Subsequently, we analyzed unstimulated lung CD4+T cells for intracellular Th1 signature transcription factor T-bet protein expression **(**Fig. 3**)**. In these studies, we observed an increased number of T-bet-expressing CD4+T cells **(**Fig. 3a**)** but not T-bet expressing CD8+T cells **(**Fig. 3b**)** in the lungs of tumor-bearing mice lacking Blimp-1 in T cells as compared to control littermates.

### Targeted deletion of Blimp1 in T cells results in induction of cytotoxic effector functions in CD8-negative lung T cells from tumor-bearing mice

We next asked if the lung cells of mice without Blimp1 in T cells would be able to better control tumor growth by inducing the cytotoxic function of lung T cells. To continue the analysis of the role of Blimp-1 in lung CD4+T cells and their role on the cytotoxicity against lung cancer cells, in this model of lung cancer, we then depleted CD8+T cells from the lung cells and challenged the remaining T cells with anti-CD3/CD28 antibodies **(**Fig. 3c**)**. We found that the absence of Blimp-1 in T cells enhanced cytotoxic function of lung CD8- T cells isolated from the lung of tumor-bearing mice against LL2 tumor cells as compared to lung CD8- T cells isolated from wild-type mice **(**Figs. 3** d).**

### Targeted deletion of Blimp-1 in T cells resulted in reduction of Foxp3+CD25+CD4+CD3+Treg cells in the lung and draining lymph nodes of tumor-bearing mice

**Fig. 4:**
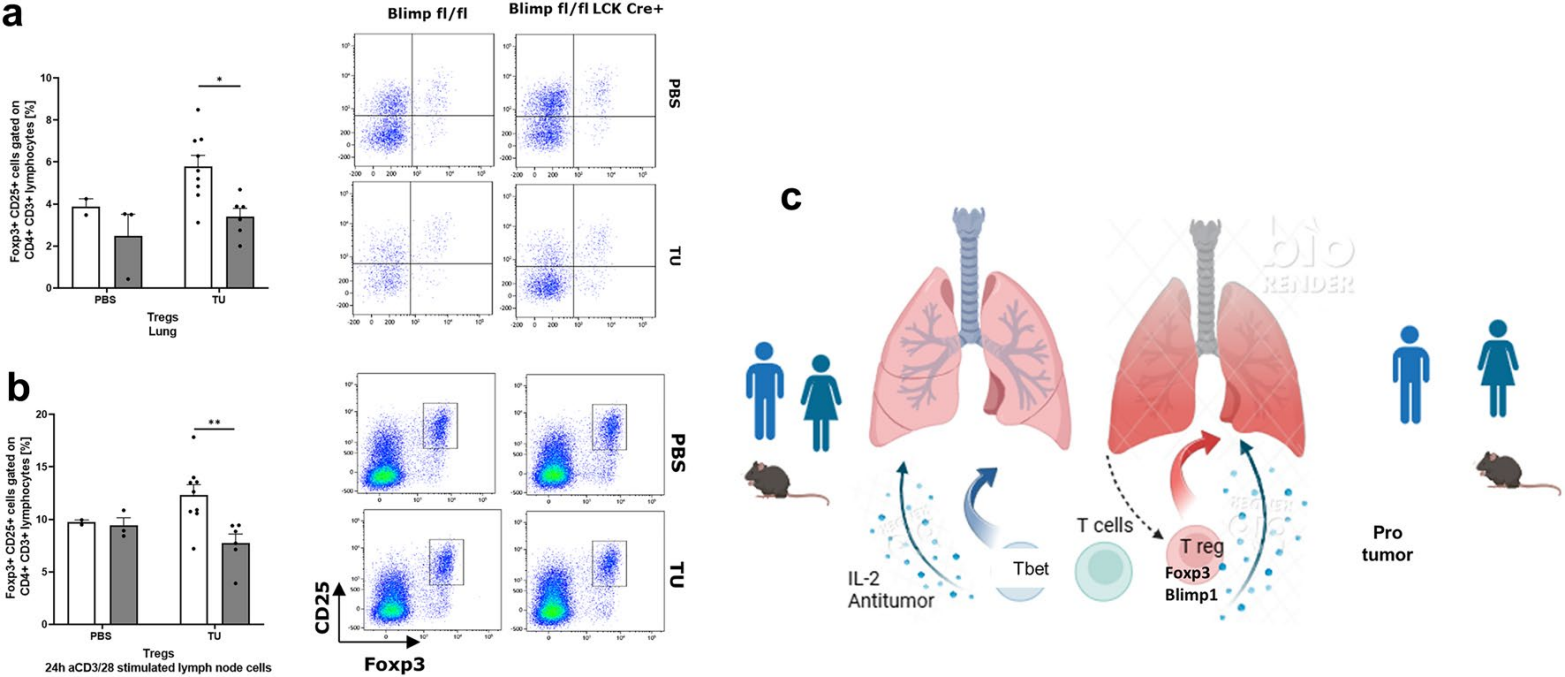
Reduction of Foxp3+T reg in the lung and lymph nodes of mice bearing tumor **a.** Flow cytometry analysis of Foxp3+CD25+Treg cells in the lung and regional lymph nodes of tumor-bearing mice (*n* = 2, 3, 9, 6). Cells were left unstimulated (panel a) or stimulated with anti-CD3/CD28 antibodies, respectively **b**. **c**. Biorender generated cartoon as a summary of the results in the lung. In this graphic summary, one can see that T-bet induction in the absence of Blimp-1 in T cells leads to IL-2-mediated anti-tumor response. By contrast the presence of Blimp in T regs leads to a pro-tumor immune response. Data are expressed as mean values ± SEM. Significance was calculated by student’s t-test or ordinary two-way ANOVA with post-hoc Šidák’s multiple comparisons test

Since we found an induction of anti-tumor function in the CD4+T cell compartment of the lung, we next asked whether this could be associated with a decrease in T regulatory immunosuppressive cells. To further demonstrate the role of Blimp1 in Treg cells, an analysis was conducted on T regulatory cells in the lungs of mice in which Blimp-1 in T cells was defective. Here, we found that the number of Foxp3⁺CD25⁺ CD4⁺CD3⁺ T regulatory cells was reduced in the lungs of tumor-bearing mice in the absence of Blimp-1 in T cells, compared to control mice (Fig. 4a, upper panel). Additionally, a reduction in the number of Treg cells after anti-CD3/CD28 activation was observed in the lymph nodes of tumor-bearing mice lacking Blimp-1 in T cells (Fig. 4b, lower panel).

In summary, targeting Blimp also resulted in an inhibition of immunosuppressive Foxp3⁺ T regulatory cells in the lungs and lymph nodes of tumor-bearing mice. These findings indicate that Blimp-1 expression plays a pivotal role in shaping the immunosuppressive environment within the tumor microenvironment in NSCLC (Fig. 4c).

Overall, our data indicate that T-bet induction in the absence of Blimp-1 in T cells leads to an IL-2- mediated anti-tumor response, whereas the presence of Blimp-1 in Tregs leads to a pro-tumor immune response.

### RNA seq analysis of CD4+and CD8+T cells from the spleen of mice lacking Blimp1 in T cells and bearing tumor show increased interferon-related gene expression

**Fig. 5:**
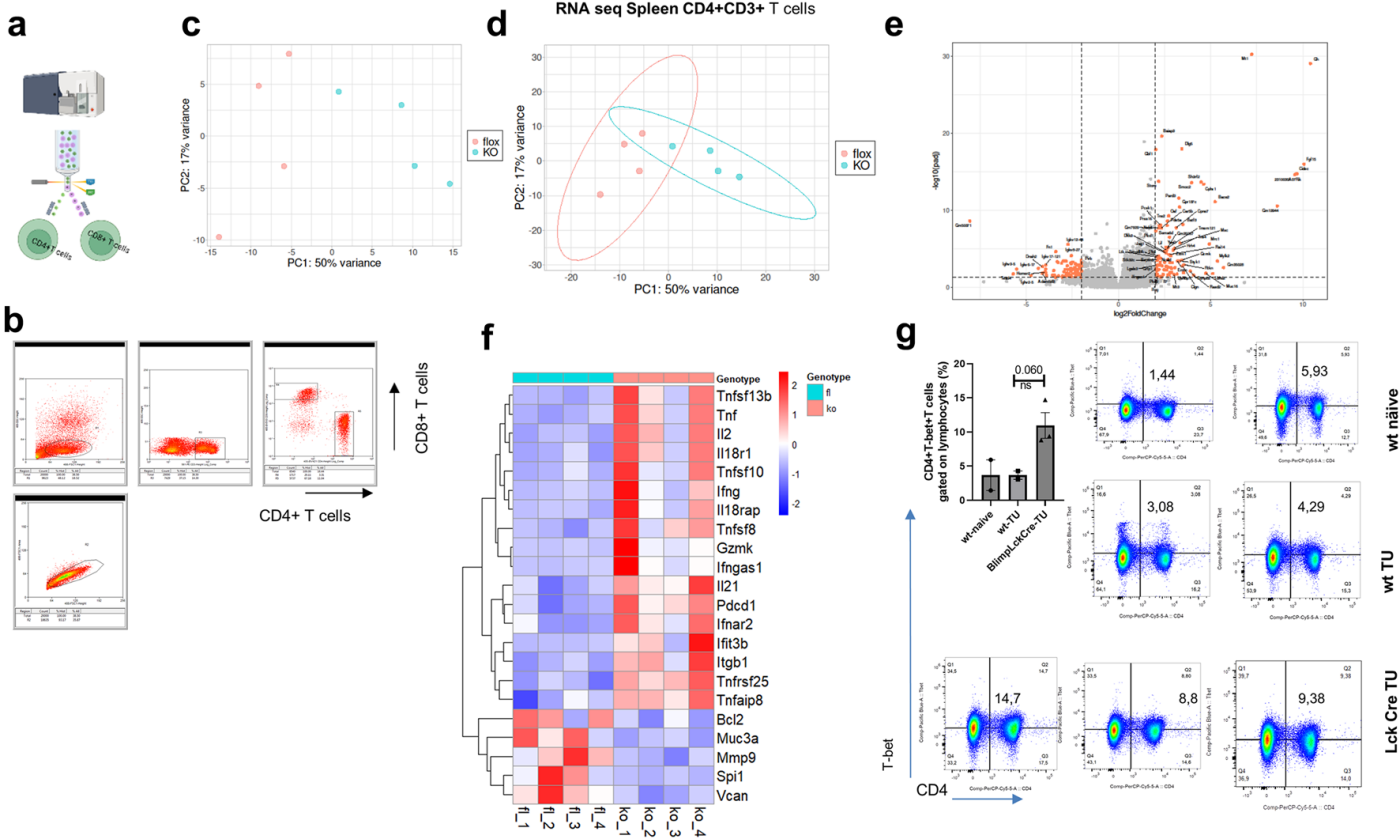
Blimp 1 deficiency in T cells results in Th1 cell expansion and induction of Th1 associated gene transcripts in the spleen. **a,b.** Spleen CD4+T-cell sorting before RNA-sequencing analysis. **b.** Representative analysis of T cells before FACS sorting. **c.** PCA Plots displaying gene expression profile clustering for Blimpfl/fl and Blimpfl/fl-Lck Cre tumor-bearing mice groups. **d.** Venn Diagram illustrating the varying gene expressions between T cells from tumor-bearing Blimp1^fl/fl^ and Blimp-1^ fl/fl^Lck Cre mice. **e.** Volcano plot identified changes in large data sets composed of replicate data by plotting significance versus fold-change on the y and x axes, respectively. **f.** Heatmap analysis showing differentially expressed genes in splenic CD4+T cells from tumor-bearing Blimp-1^ fl/fl^ and Blimp-1^ fl/fl^xLckCre mice bearing tumor (*n* = 3/4 per group). Genes with significant differences in expression (logFC > 1.5 or < −1.5, padj < 0.05) between the groups are highlighted. **g.** FACS of CD4+T-bet+splenic T cells in wild-type and conditional deficient mice. Unpaired T-test. Data are shown as mean values ± SEM

We next extracted RNA and performed RNA-sequencing analysis for spleen CD4+T cells from wild- type and Blimp1LckCre mice bearing tumor **(**Figs. 5a–f**)**. Our findings revealed that numerous genes with anti-tumoral function were regulated by Blimp-1 in T cells. In the absence of Blimp-1 in CD4+T cells, the expression of IFN-gamma and IFN-related genes was upregulated, as well as that of cytotoxic genes. This finding suggests that Blimp-1 suppresses the Th1 functions of CD4+T cells in experimental NSCLC. Here, we observed that PD1 expression (Pdcd1) was induced in CD4+T cells lacking Blimp-1 in comparison to wild-type control cells [11]. Furthermore, T-bet protein expression was quantified and found to be induced in CD4+T cells in the absence of Blimp1, in T cells **(**Fig. 5g**)**. Thus, we conclude that the anti-tumoral function of T-bet can overcome the presence of PD1 in the absence of Blimp-1 in T cells. Further experiments are underway and are needed to fully demonstrate this.

We next analyzed isolated CD4+and CD8+T cells from the spleen of wild-type and T-bet deficient mice **(Figure S3a-c)** and skewed these T cells under Th1 and Tc1 conditions. We then measured Blimp1 mRNA expression after anti-CD3/CD28 antibody stimulation in cell culture. We found that in the absence of T-bet and after anti-CD3/CD28 stimulation both CD4+and CD8+T cells had induced Blimp-1 mRNA expression **(Figure S3b).** These data indicate also an inhibitory function of T-bet on Blimp-1 expression. Moreover, FACS analysis of Th1 skewed spleen T cells showed an induction of Treg cells expressing Foxp3 and PD1 simultaneously **(Figure S3c)**, confirming our previous findings on a role of T-bet on immunosuppressive Tregs [2]. In conclusion, T-bet deficiency, a condition linked to lung tumor development [3, 4], was found to be associated to increase Blimp-1 expression in both CD4+and CD8 +T cells.

**Fig. 6:**
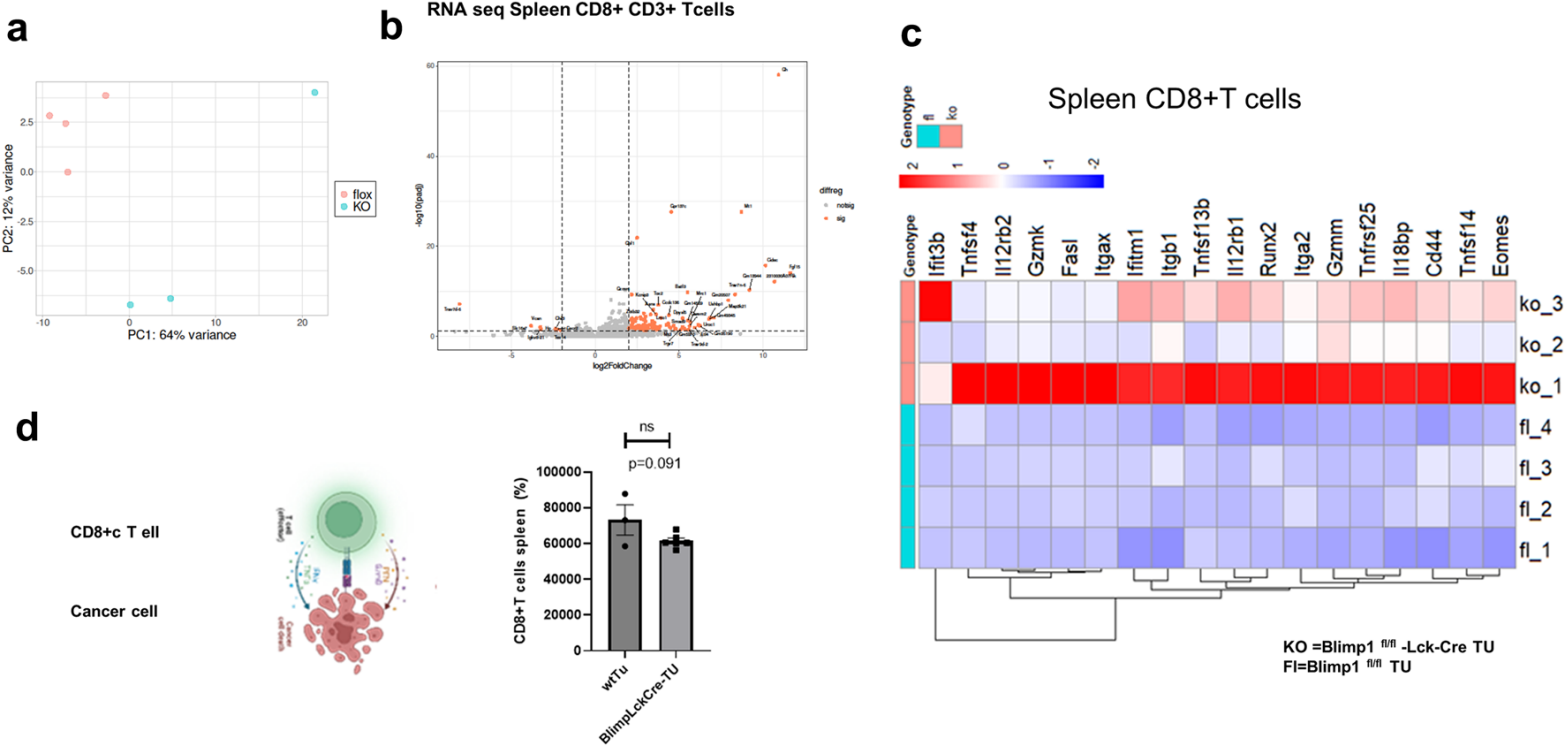
Blimp 1 deficiency in T cells results in induction of Tc1 associated gene transcripts **a**. RNA seq analysis from sorted spleen CD8+T cells isolated from Blimp^fl/fl^ and Blimp^fl/fl^LckCre Tumor-bearing mice. Spleen CD8+T cells were sorted by FACS from Blimp^fl/fl^ and Blimp^fl/fl^/LckCre+tumor-bearing mice**.** RNA-seq experimental design for sorted splenic CD8+T cells from tumor-bearing mice is shown. **b.** Volcano plotting identified changes in large data sets composed of replicate data by plotting. Significance versus fold-change on the y and x axes, respectively. **c.** Heatmap representing significant differentially expressed genes in splenic CD8+T cells from tumor-bearing Blimp1^fl/fl^ mice versus Blimp1^fl/fl^ xLckCre mice bearing tumor (*n* = 3/4 per group). The different groups are indicated. Genes exhibiting notable differential expression (logFC > 1.5 or < −1.5, padj < 0.05) between the groups are highlighted. **d**. The supernatants of spleen CD8+T cells isolated from the mice with tumor were analyzed diluted to 30% in this cytotoxic assay. Data were analyzed using the Mann–Whitney test or the unpaired two-tailed T-test, and are presented as mean values ± SEM

Regarding CD8+ T cell RNA-seq analysis, it was observed that the absence of Blimp-1 resulted in a clear upregulation of IFN-gamma and type I interferon gene expression as well as an induction of cytotoxic genes in CD8+T cells when compared to controls (Fig. 6 a–c). Moreover, spleen CD8+T cells from Blimp1-deficient mice exhibited enhanced cytotoxic function against the tumor cell line utilized to induce tumors in mice **(**Figs. 6d**)**, suggesting that Blimp-1 plays a pivotal role in regulating cytotoxic effector functions of T cells in NSCLC.

## Discussion

In this manuscript, we set out to investigate the role of Blimp1, a transcriptional repressor, in T cells in a murine model of lung cancer.

We found that targeting Blimp1 in T cells resulted in decreased lung cancer and increased effector cytotoxic function of T cells in the lung and periphery. This effector function was dominated by induction of the transcription factor T-bet. Furthermore, we found an inhibition of T regulatory cells bearing Foxp3 as a transcription factor signature in the lungs and local lymph nodes of tumor-bearing mice lacking Blimp1 in T cells.

Although a common role of T-bet and Blimp1 in CD8⁺ T cells has been described, their role there was not entirely clear [12]. In this study, we show that targeting Blimp1 in T cells resulted in expansion of T-bet⁺ CD4⁺ T cells in the lungs of tumor-bearing mice. Thus, these data indicate a repressive function of Blimp1 on T-bet in CD4⁺ T cells. This regulatory function could be important for tumor cell grwoth, as we have previously demonstrated that T-bet deficiency results in induction of lung cancer in mice and humans [2, 3, 13, 14].

Moreover, here we show that T-bet deficiency is associated with Blimp-1 induction under Th1-skewing conditions, demonstrating that T-bet can also repress Blimp-1. Thus, targeting Blimp-1 in T cells would result in increased effective T cells in the lungs of tumor-bearing mice. This point was demonstrated using different methods, showing that Blimp1 deficiency in T cells resulted in upregulation not only of T-bet but also of T-bet-regulated interferon genes. In fact, we performed FACS staining, RNA-seq analysis, as well as ELISA and multiplex cytokine assays.

Moreover, this increase in the interferon pathway was accompanied by a decrease in Foxp3⁺ T regulatory cells in the lungs and lymph nodes of tumor-bearing mice. There are reports demonstrating that IFN-γ induces Treg instability [15]. We recently demonstrated a considerable accumulation of T-bet⁺Foxp3⁺CD4⁺ T cells, mediated by the immunosuppressive cytokine TGF-β, in the lungs of tumor-bearing mice [2].

By integrating results from both murine models and human disease, we demonstrate that the conversion of IFN-*γ*-producing anti-tumoral T-bet⁺ Th1 CD4⁺ T cells into immunosuppressive T-bet⁺Foxp3⁺PD1⁺ regulatory cells could represent an additional important mechanism of TGF-*β*-mediated blockade of anti-tumor immunity [2].

In addition, in this paper, we report the induction of Foxp3⁺PD1⁺ cells in spleen cells from T-bet-deficient mice skewed under Th1 conditions, whereby Blimp-1 was also upregulated in T-bet-deficient cells skewed into Th1, along with Tregs expressing PD1. Moreover, Blimp-1 is also present in T regulatory cells, where it antagonizes factors that destabilize Tregs. Taken together, we propose that in Foxp3⁺ Tregs, Blimp1 might antagonize T-bet. This concept aligns with the finding that targeted deletion of PD1 resulted in destabilization of Tregs [16–18]

In conclusion, targeting Blimp1 thus would have a beneficial effect on anti-tumor response by upregulating Th1 and Tc1 cells. This was also confirmed by RNA seq data in CD4+and CD8+T cells from the Blimp-1 conditional knockout mice. In fact, in CD4+T cells isolated from the spleen of Blimp1 conditional knockout mice, we found an upregulation of Th1 related factors like Ifngas1, IL-2, IL-18r1, IFng, and Gzmk. Similarly, in CD8+T cells, we found the same trend toward upregulation of Tc1-related genes. This skewed phenotype was not as strong as in the CD4+T cell compartment, however. By observing the function of T cells, we looked at the cytotoxic function against the tumor cell line used to induce tumors in mice. In these studies, we found an increased cytotoxicity in the absence of Blimp-1 only in the lung cells depleted from CD8+T cells stimulated with anti-CD3 and anti-CD28 antibodies in the absence of Blimp-1. Moreover, we found induction of cytotoxic factors in the spleen CD8+T cell compartment in mice bearing tumor and lacking Blimp-1 in T cells. Remarkably, 30% of the supernatant resulted in tumor cell death indicating that soluble factors like IFN*γ* (which was measured) can inhibit tumor growth. Further experiments using cell to cell contact induced cell death could also improve our knowledge on how Blimp1 deficiency modifies T cells to make them more effective at inhibiting tumor cells through direct interactions.

Moreover, another limiting aspect of the present study is that the LckCre promoter drives Cre expression early in thymocyte development, leading to Blimp-1 deletion in all T cells from the earliest stages of maturation. This model prevents the analysis of Blimp-1 ‘s role specifically in mature T cells or in the TME. It is possible that early deletion may also affect T cell development or stability outside of the cancer context.

In conclusion, Blimp1 deficiency could offer the possibility to target T regulatory cells and induce anti-tumor response in patients via upregulation of T-bet in CD4+T cells. Moreover, it could also improve the CD8+T-cell repertoire, making it more effective against the tumor.

## Supplementary Information

Below is the link to the electronic supplementary material.

Supplementary file1 (DOCX 745 KB)

## Data Availability

No datasets were generated or analysed during the current study.
