# Supplementary material for: Targeting Blimp-1 in T cells results in activation of T-bet-mediated control of immunosuppression in lung cancer
Source: Cancer Immunol Immunother. 2026 Apr 15;75(5):144. doi: 10.1007/s00262-026-04381-4 (PMC13083746; doi:10.1007/s00262-026-04381-4)
Supplement: Supplementary file 1 — Supplementary file1 (DOCX 745 KB) [file 262_2026_4381_MOESM1_ESM.docx]

Supplementary DATA

**Targeting Blimp-1 in T cells resulted in activation of T-bet mediated control of immunosuppression in lung cancer**

Susetta Finotto^1,2,3,4*^, Mircea Chiriac^5^, Susanne Krammer^1^, Zuqin Yang^1^, Laura Neurath^1^, Carol I. Geppert^6,3,4^, Sonja Trump^1^, Adriana Geiger^1^, Elvedina Nendel^1^, Stefan Wirtz^5^, Sebastian Zundler^2,5^ and Markus F. Neurath^5,2,3,4^

^1^ Department of Molecular Pneumology, Friedrich Alexander University Erlangen-Nürnberg (FAU), Universitätsklinikum Erlangen, 91054 Erlangen, Germany.

^2^ Deutsches Zentrum für Immuntherapie (DZI), Erlangen, Germany,

^3^ Bavarian Cancer Research Center (BZKF), Erlangen, Germany,

^4^ Comprehensive Cancer Center Erlangen-EMN (CCC ER-EMN), Erlangen, Germany

^5^ Department of Internal Medicine 1, Friedrich Alexander University Erlangen-Nürnberg (FAU), Universitätsklinikum Erlangen, 91054 Erlangen, Germany.

^6^ Institute of Pathology, University Hospital, Friedrich-Alexander-Universität Erlangen-Nürnberg, Erlangen, Germany

^7^ Department of Thoracic Surgery, University Hospital, Friedrich-Alexander-Universität Erlangen-Nürnberg, Erlangen, Germany

^8^ Department of Immune Modulation, Dermatology Clinic, Friedrich Alexander University Erlangen-Nürnberg (FAU), Universitätsklinikum Erlangen, 91054 Erlangen, Germany

***Correspondence:**

Prof. Dr. Dr. Susetta Finotto

Universitätsklinikum Erlangen

Abt. Molekulare Pneumologie

Hartmannstraße 14

91052 Erlangen

Phone: +49-9131-85-35883

[Mail: susetta.finotto@uk-erlangen.de](mailto:susetta.finotto@uk-erlangen.de)

[http://www.molekulare-pneumologie.uk-erlangen.de](http://www.molekulare-pneumologie.uk-erlangen.de/)

“The authors declare no competing interests.”

## Table S1: List of antibodies used for Flow Cytometry.

| **Antigene** | **Conjugate** | **Reactivity** | **Dilution** | **Company** | **Cat#** |
| --- | --- | --- | --- | --- | --- |
| CD3 | PE-Cy7 | Murine | 1:200 | BD Biosciences | 552774 |
| CD4 | PerCP-Cy5.5 | Murine | 1:200 | BD Biosciences | 550954 |
| CD8a | PerCP-Cy5.5 | Murine | 1:150 | BioLegend | 100734 |
| CD8a | BV510 | Murine | 1:150 | BD Biosciences | 563068 |
| CD8a | BV421 | Murine | 1:150 | BD Biosciences | 563898 |
| CD25 | APC-Fire750 | Murine | 1:100 | BioLegend | 102053 |
| CD25 | APC-Cy7 | Murine | 1:100 | BD Biosciences | 557658 |
| CD279 | PE | Murine | 1:100 | BD Biosciences | 561788 |
| CD279 | BV510 | Murine | 1:100 | BioLegend | 135241 |
| CD279 | BV711 | Murine | 1:100 | BioLegend | 135231 |
| FoxP3 | AlexaFluor 647 | Murine | 1:50 | BioLegend | 126408 |
| T-bet | BV421 | Murine | 1:50 | BD Biosciences | 563318 |

## Supplementary Table S2: List of primers used for Quantitative Real-Time PCR.

| Species | Gene | Primer Sequence Eurifins Genomic |
| --- | --- | --- |
| Mouse | *Hprt* | For: 5´-GCC CCA AAA TGG TTA AGG TT-3`  Rev: 5′-TTG CGC TCA TCT TAG GCT TT-3` |
|  | *Tbet* | For: 5`-CCA CCT GTT GTG GTC CAA GTT C-3` |
|  | *Prdm1* | BioRad Cat n . 10025636 |

**Supplementary Figures**

**
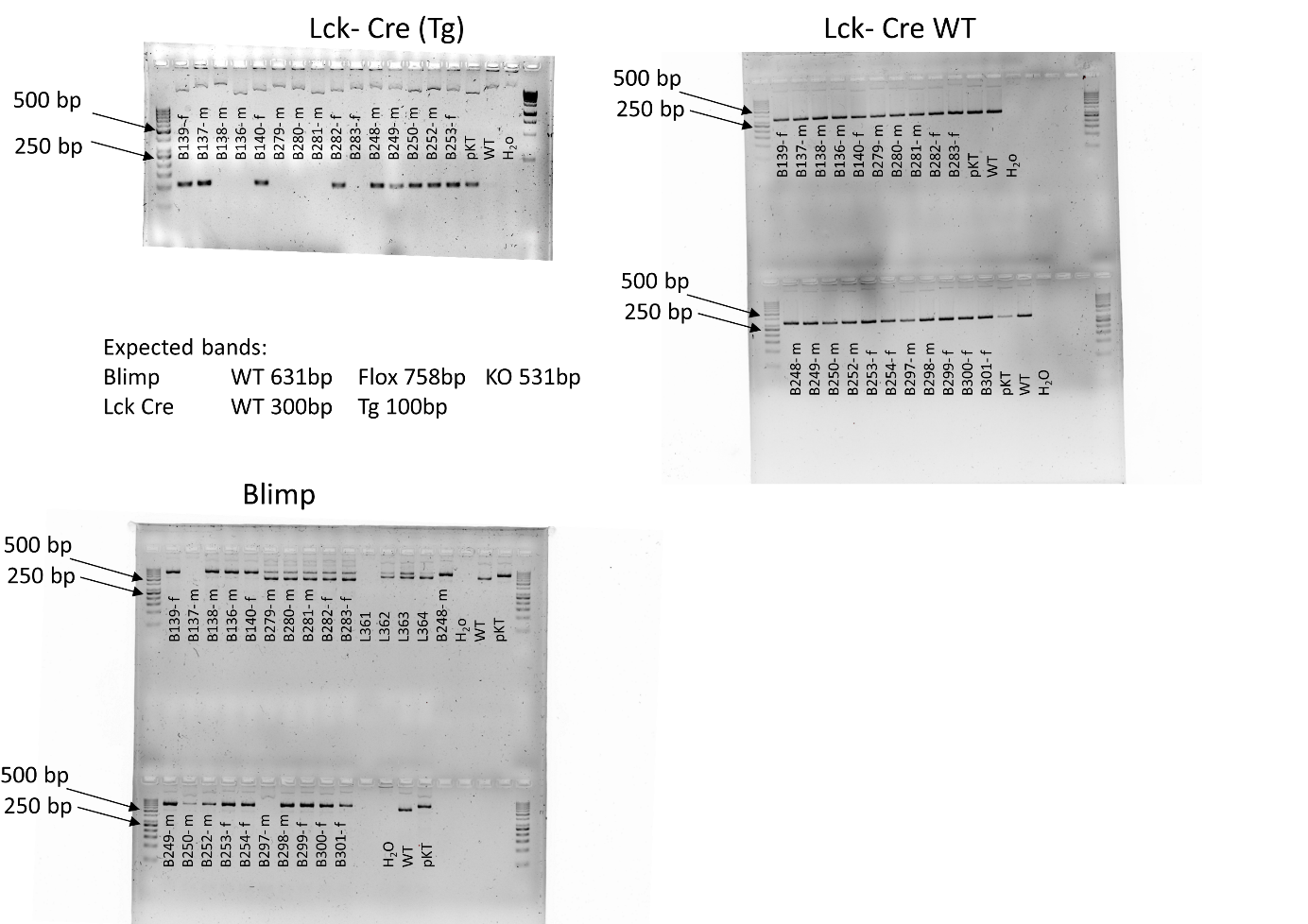
**

**Supplementary Figure 1. Genotyping of the mice used in this study.**

**
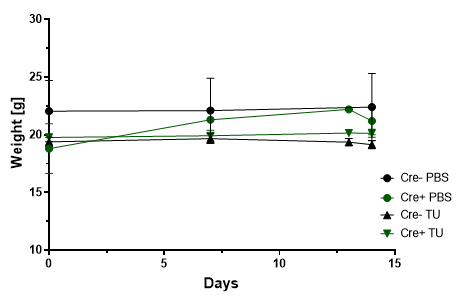
**

**Supplementary Figure 2:**  Weight measurements during the experimental model of lung cancer.


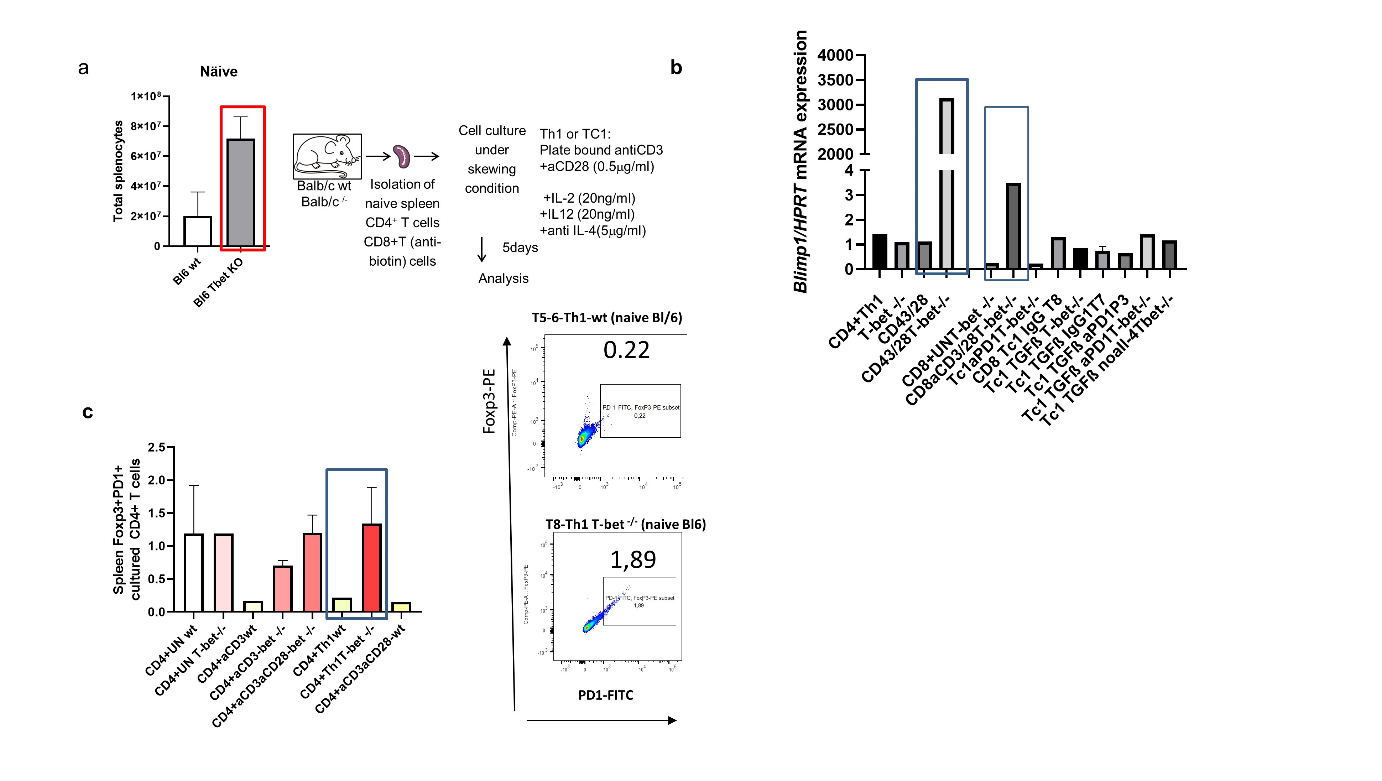


**Supplementary Figure 3. T cells from mice lacking T-bet induced Blimp-1 after antiCD3CD28 stimulation. a.** Experimental design of Tc1 and Th1 skewing conditions in spleen cells from wt and T-bet deficient mice. CD4+ and CD8+ cells were sorted with magnet beads bound antibodies and skewed under Tc1 and Th1 skewing conditions.**b.** RNA was extracted after cell culture and qPCR Blimp1/HPRT mRNA by qPCR was performed in n=2 per group. **c.** FACS analysis of T regs Fox3+ and PD1+ CD4+ T cells. n=2 per group.
